# Supplementary material for: RARβ Expression in Keratinocytes from Potentially Malignant Oral Lesions: The Functional Consequences of Re-Expression by De-Methylating Agents
Source: Cancers (Basel). 2021 Aug 12;13(16):4064. doi: 10.3390/cancers13164064 (PMC8391937; doi:10.3390/cancers13164064)
Supplement: Supplementary file 1 [file cancers-13-04064-s001.zip › cancers-1305440 revised supp.pdf]

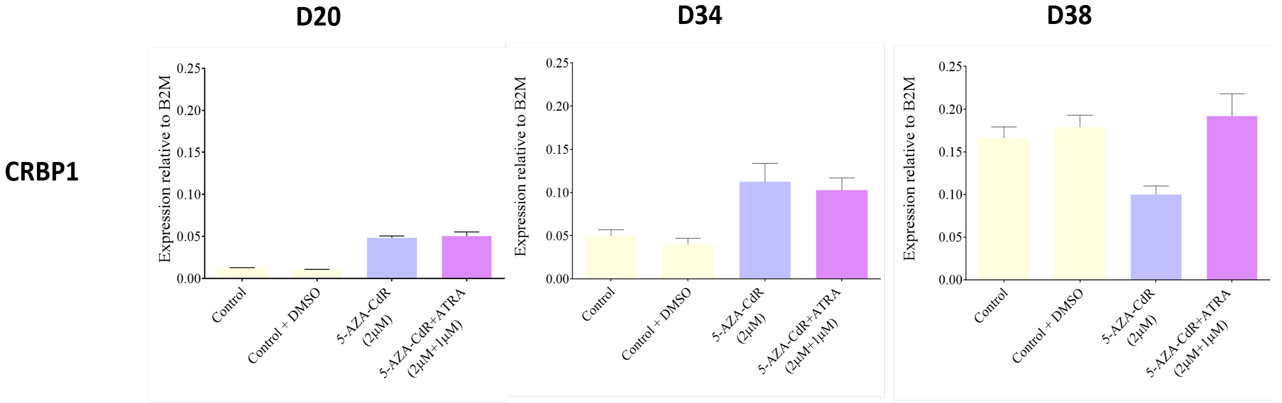

Figure S1: Expression of cRBP1 under AZA-C and ATRA treatment as assessed by qPCR. The pattern of expression in D19 is very similar to that in D34.
