# Supplementary material for: RARβ Expression in Keratinocytes from Potentially Malignant Oral Lesions: The Functional Consequences of Re-Expression by De-Methylating Agents
Source: Cancers (Basel). 2021 Aug 12;13(16):4064. doi: 10.3390/cancers13164064 (PMC8391937; doi:10.3390/cancers13164064)
Supplement: Supplementary file 1 [file cancers-13-04064-s001.zip › Original blots and gels - all figures revised.pdf]

Original blots for figure 1B – various exposures, with densitometry relative to B actin.

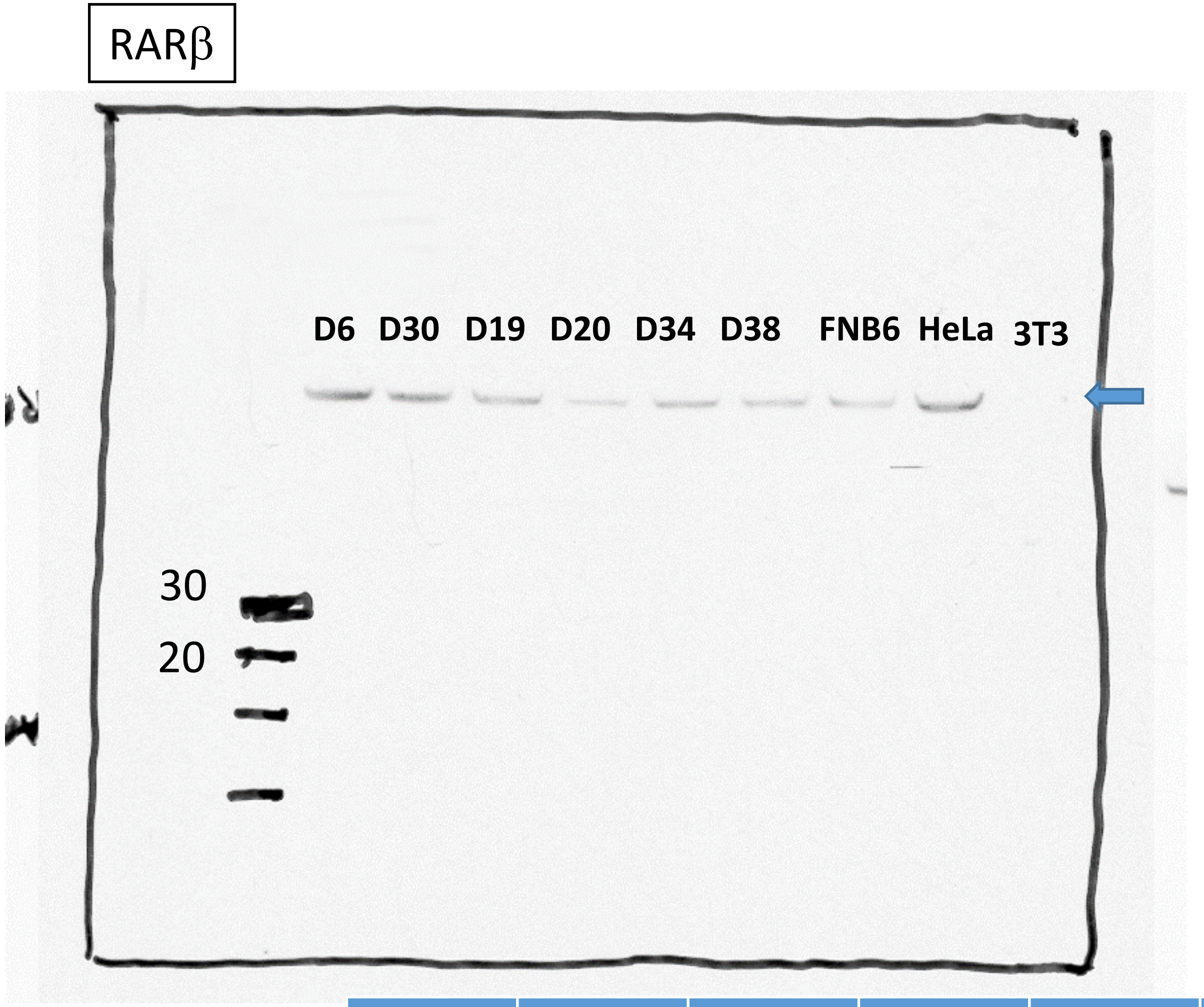


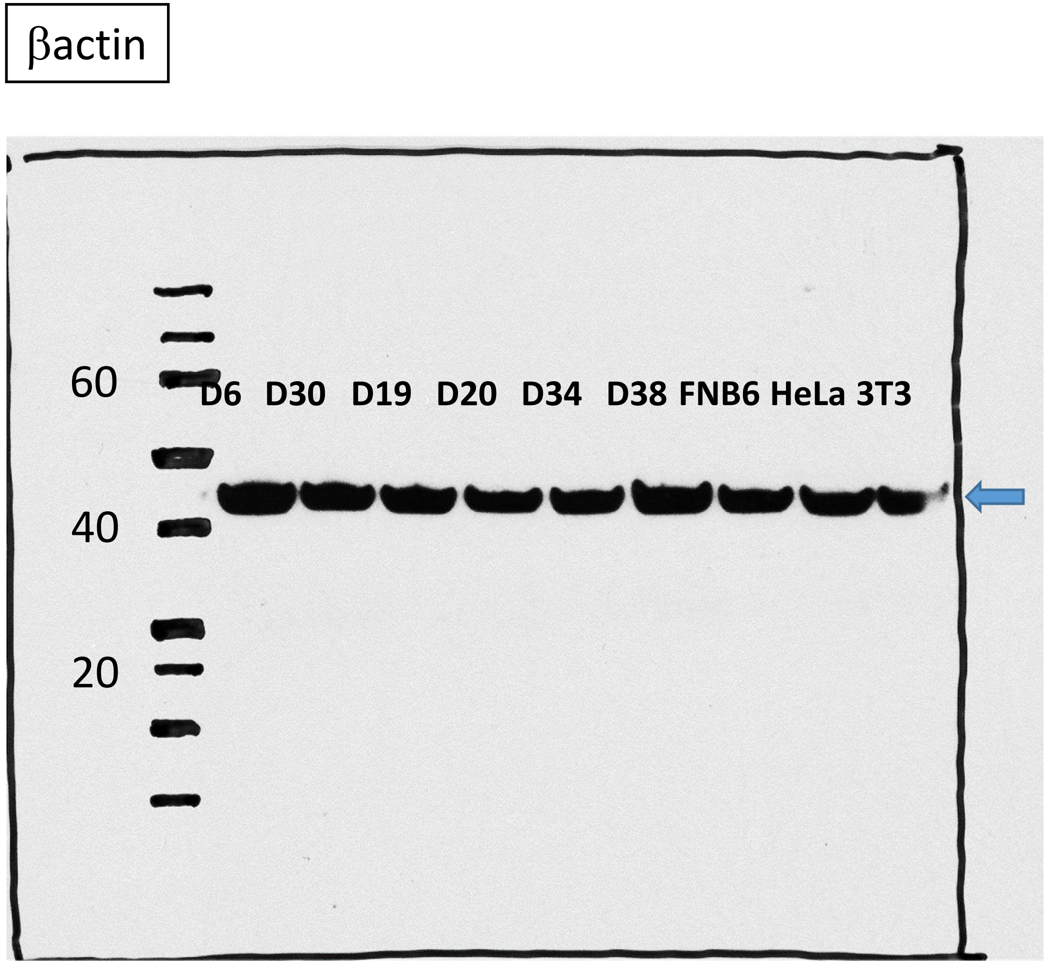


| | D6 | D30 | D19 | D20 | D34 | D38 | FNB6 | HeLa | i3t3 |
|---|---|---|---|---|---|---|---|---|---|
| RARB | 0.14 | 0.13 | 0.08 | 0.04 | 0.07 | 0.05 | 0.05 | 0.17 | 0.01 |

# Original blots for figure 1B ctd, with densitometry relative to B actin

p16

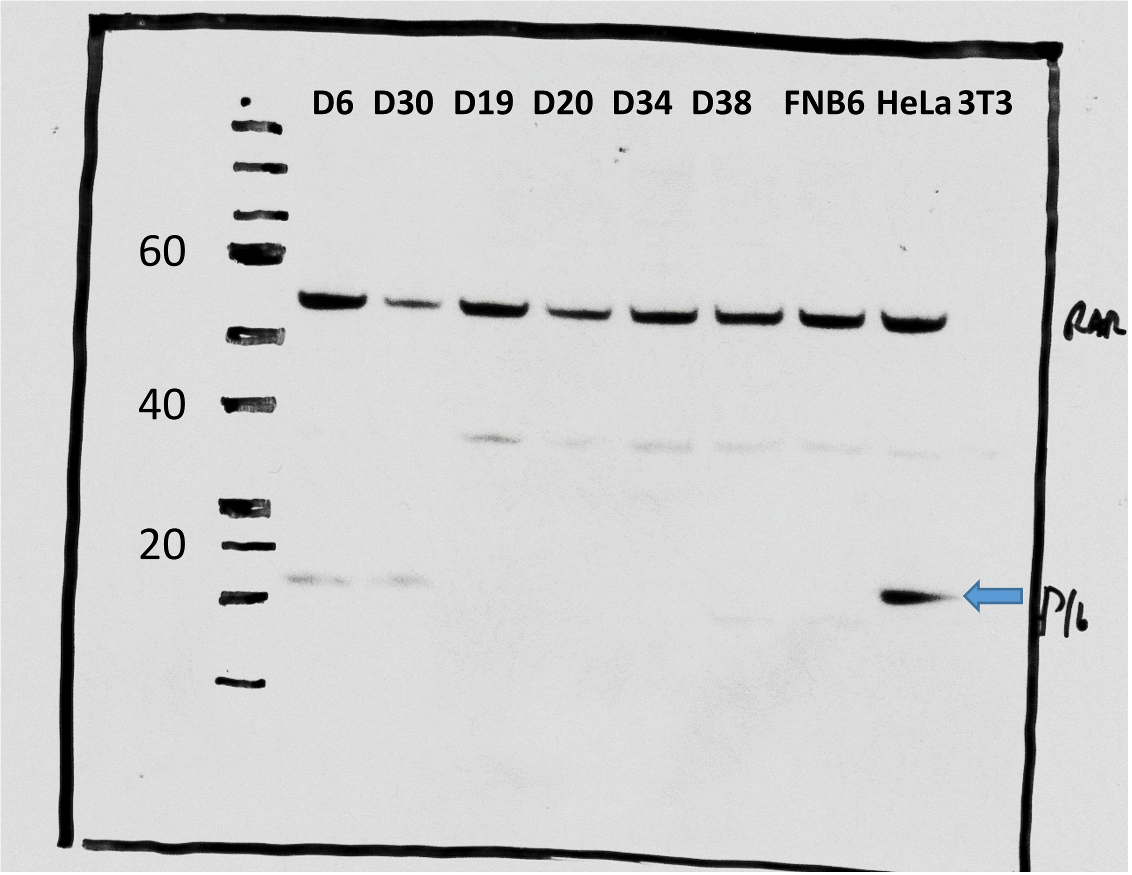


p21

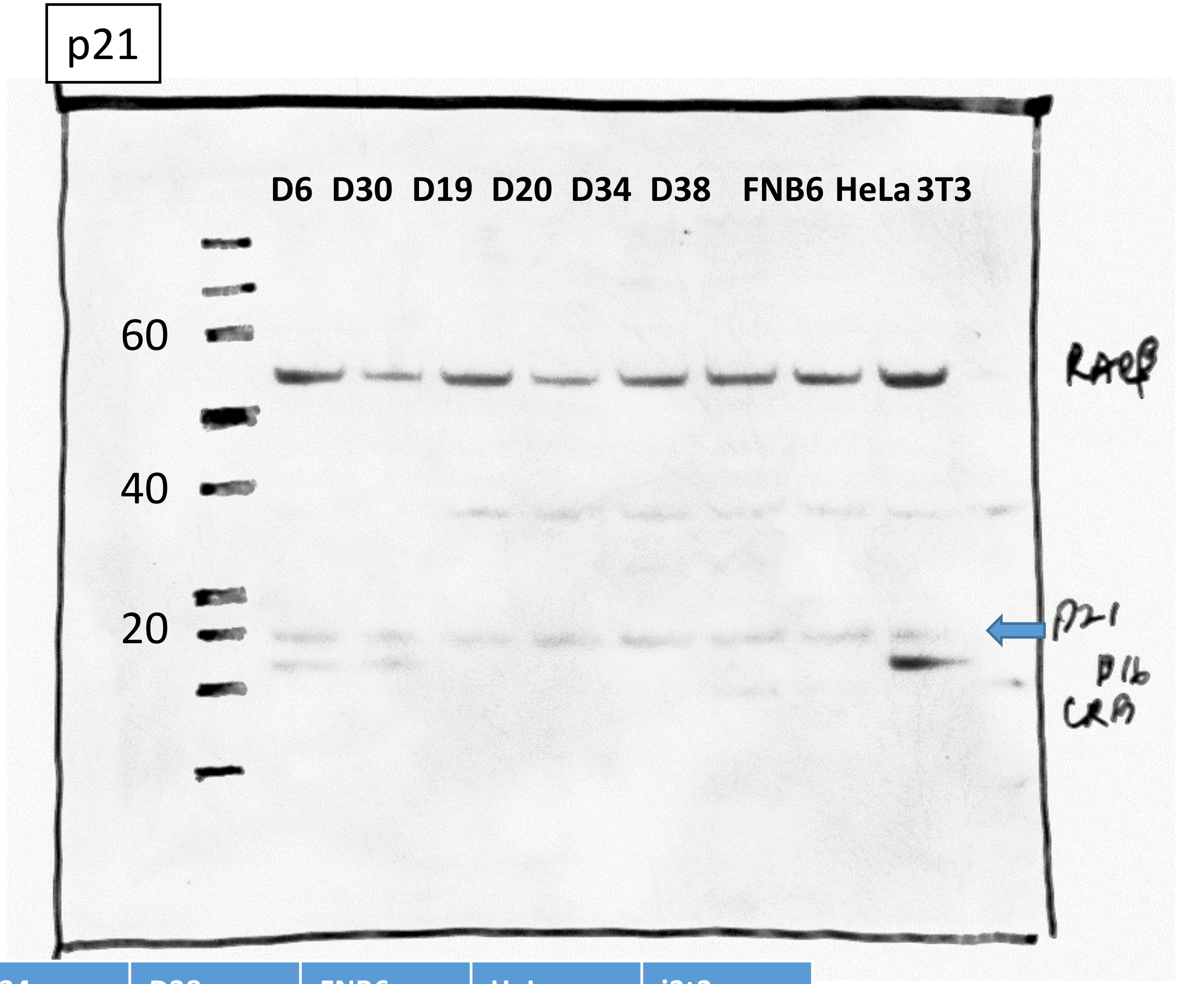


| | D6 | D30 | D19 | D20 | D34 | D38 | FNB6 | HeLa | i3t3 |
|---|---|---|---|---|---|---|---|---|---|
| p16 | 0.07 | 0.05 | 0.02 | 0.02 | 0.01 | 0.01 | 0.02 | 0.41 | 0.01 |
| p21 | 0.05 | 0.04 | 0.06 | 0.03 | 0.08 | 0.06 | 0.08 | 0.06 | 0.01 |

# Original gels for figure 2.

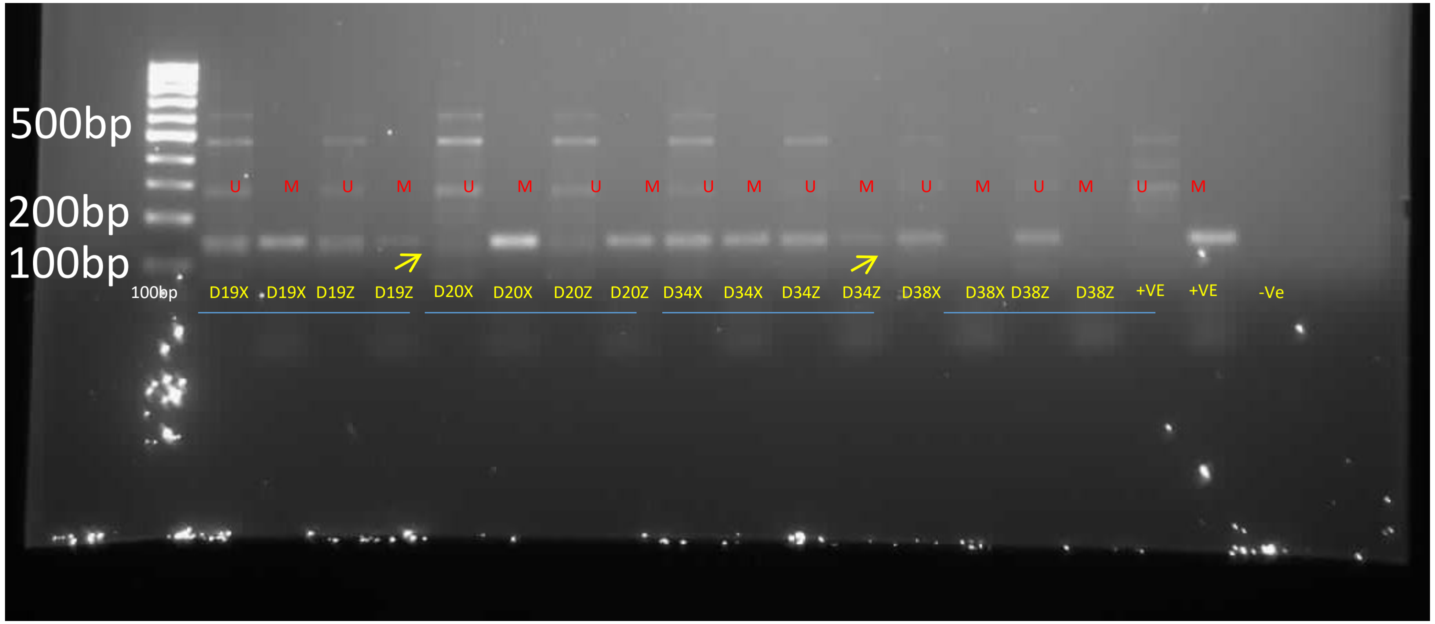

# Original gels for figure 2

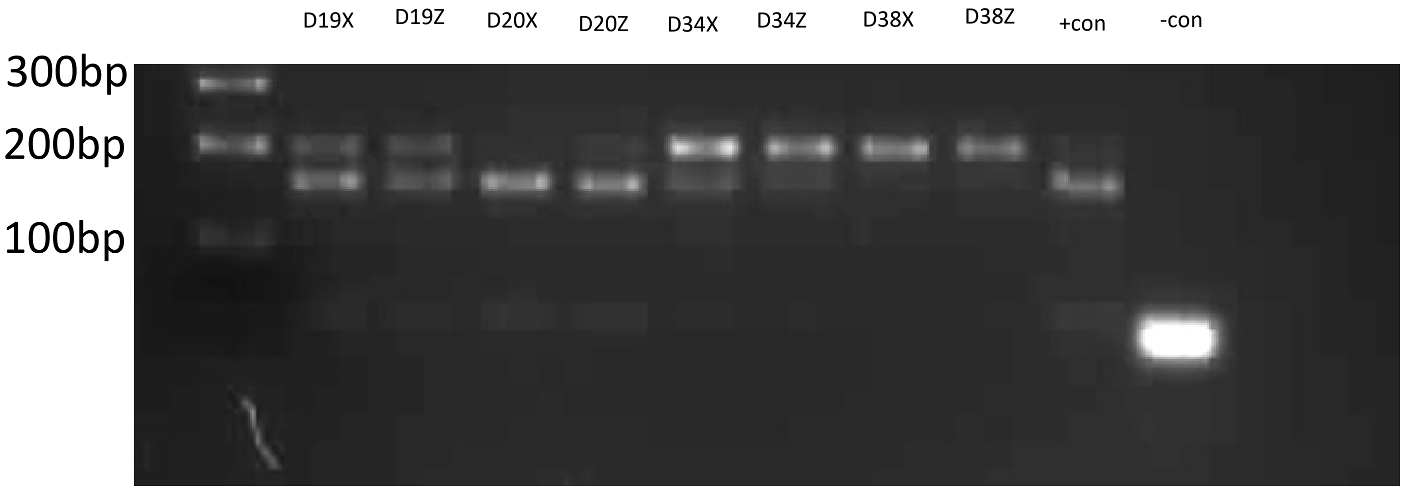

# Original blots for figure 3B: D19, with densitometry relative to B actin

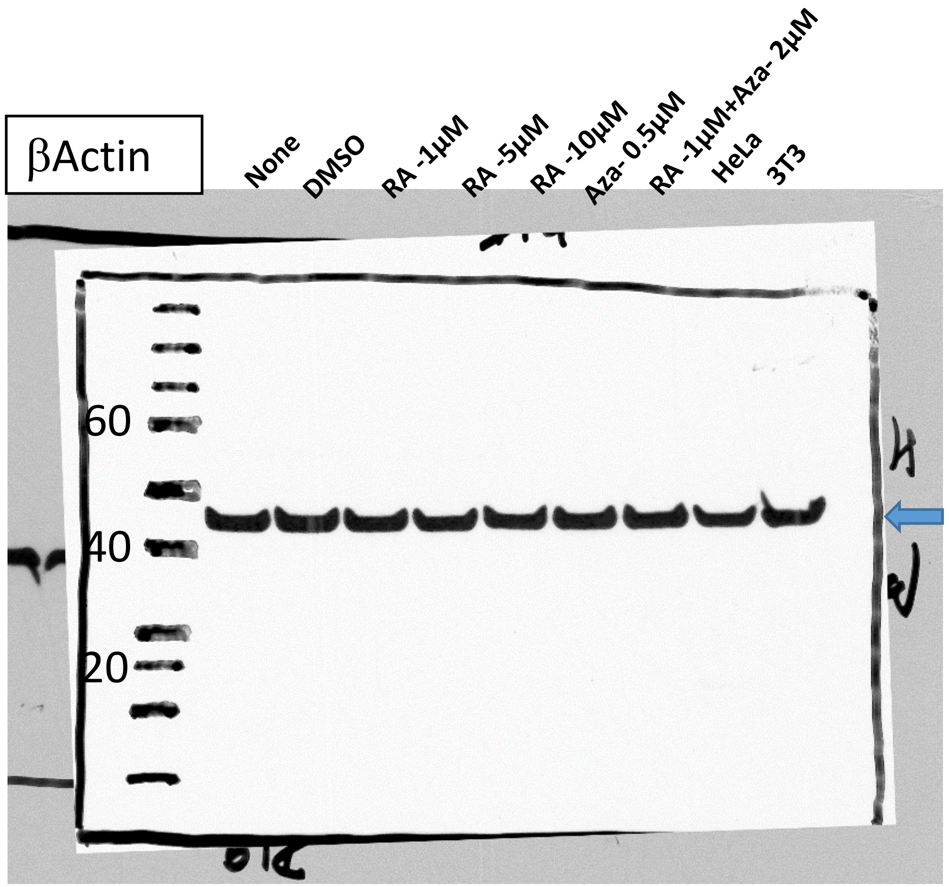


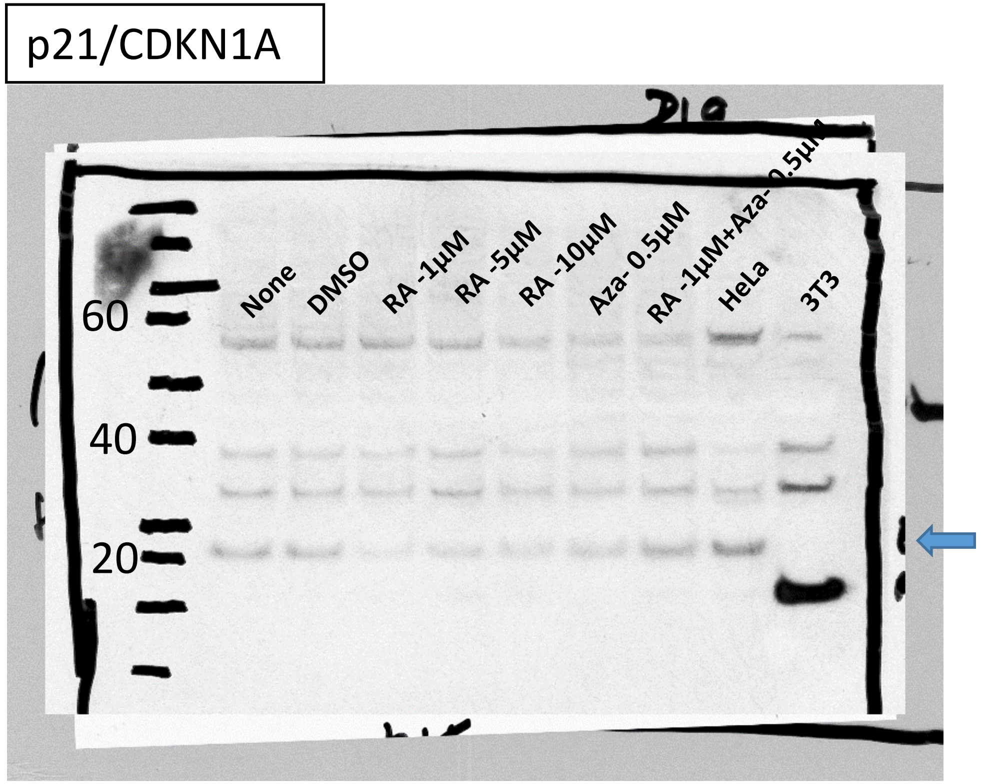


| | DMSO | No Rx | RA 1uM | RA 5uM | RA10uM | Aza 0.5uM | RA+Aza | HeLa | i3t3 |
|---|---|---|---|---|---|---|---|---|---|
| p21 | 0.13 | 0.10 | 0.03 | 0.07 | 0.07 | 0.10 | 0.18 | 0.31 | 0.00 |

# Original blots for figure 3B: D19, with densitometry relative to B actin

RARβ

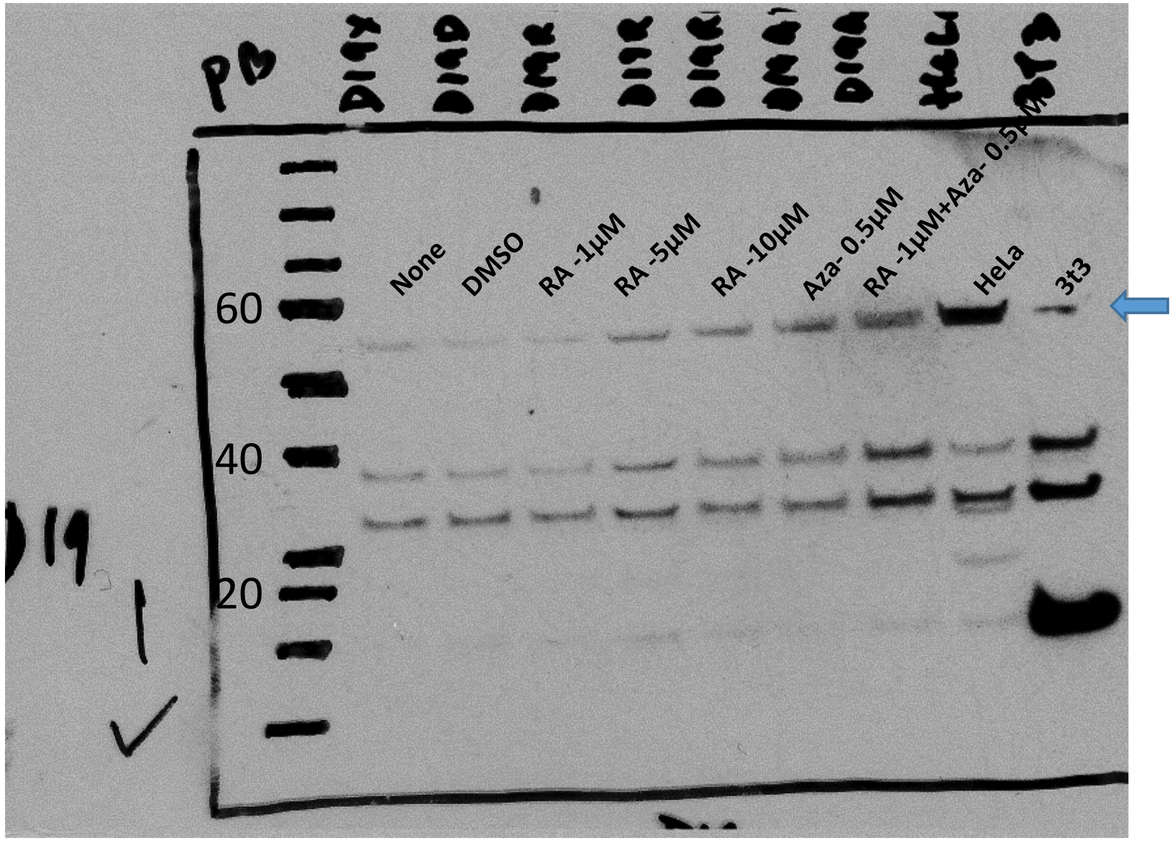


p16/CDKN2A

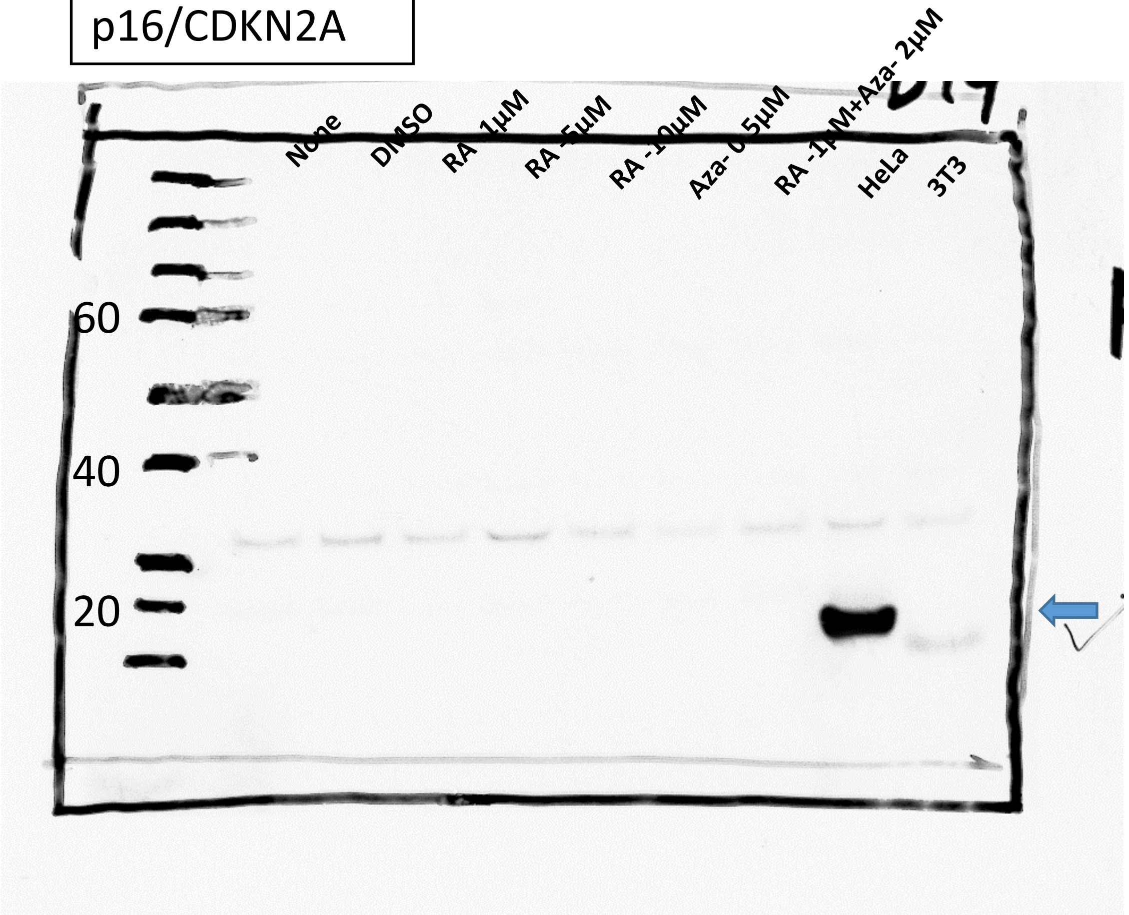


| | No Rx | DMSO | RA 1uM | RA 5uM | RA10uM | Aza 0.5uM | RA+Aza | HeLa | i3t3 |
|---|---|---|---|---|---|---|---|---|---|
| RARB | 0.02 | 0.01 | 0.01 | 0.04 | 0.04 | 0.06 | 0.07 | 0.39 | 0.03 |
| p16 | 0.00 | 0.00 | 0.00 | 0.00 | 0.00 | 0.00 | 0.00 | 0.94 | 0.00 |

# Original blots for figure 3B: D20

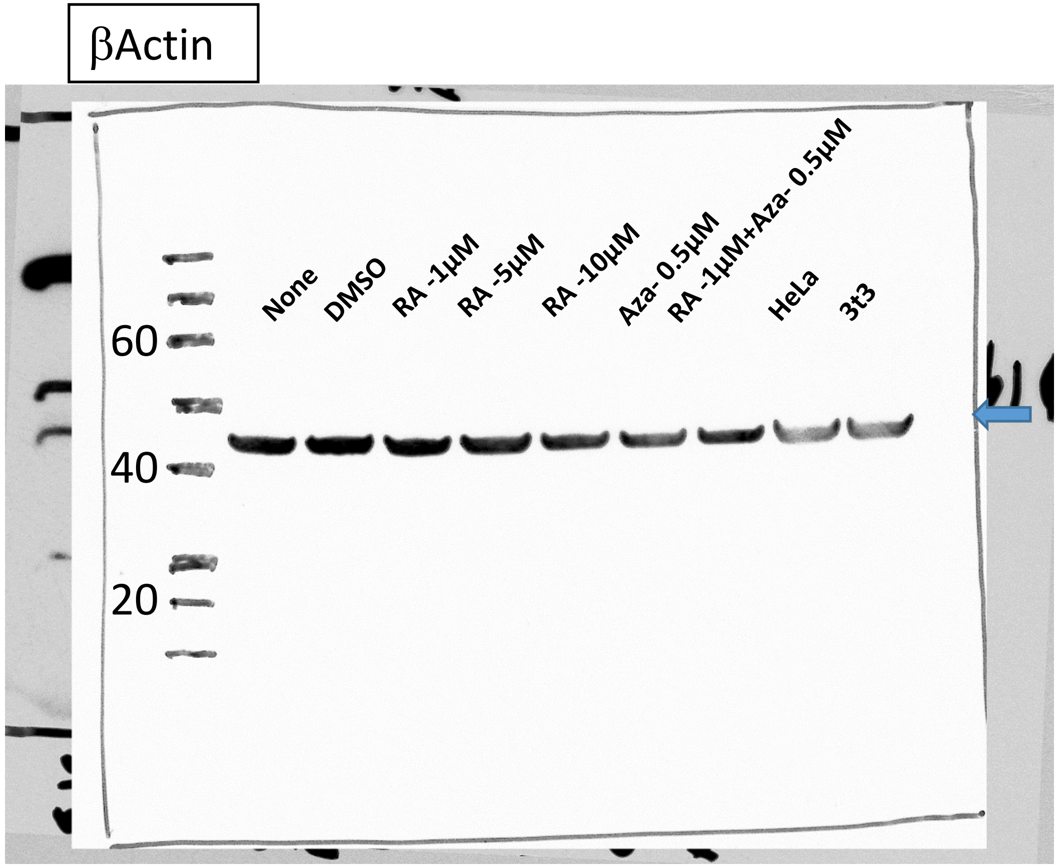

# Original blots for figure 3B: D20, with densitometry relative to B actin

RARβ p16

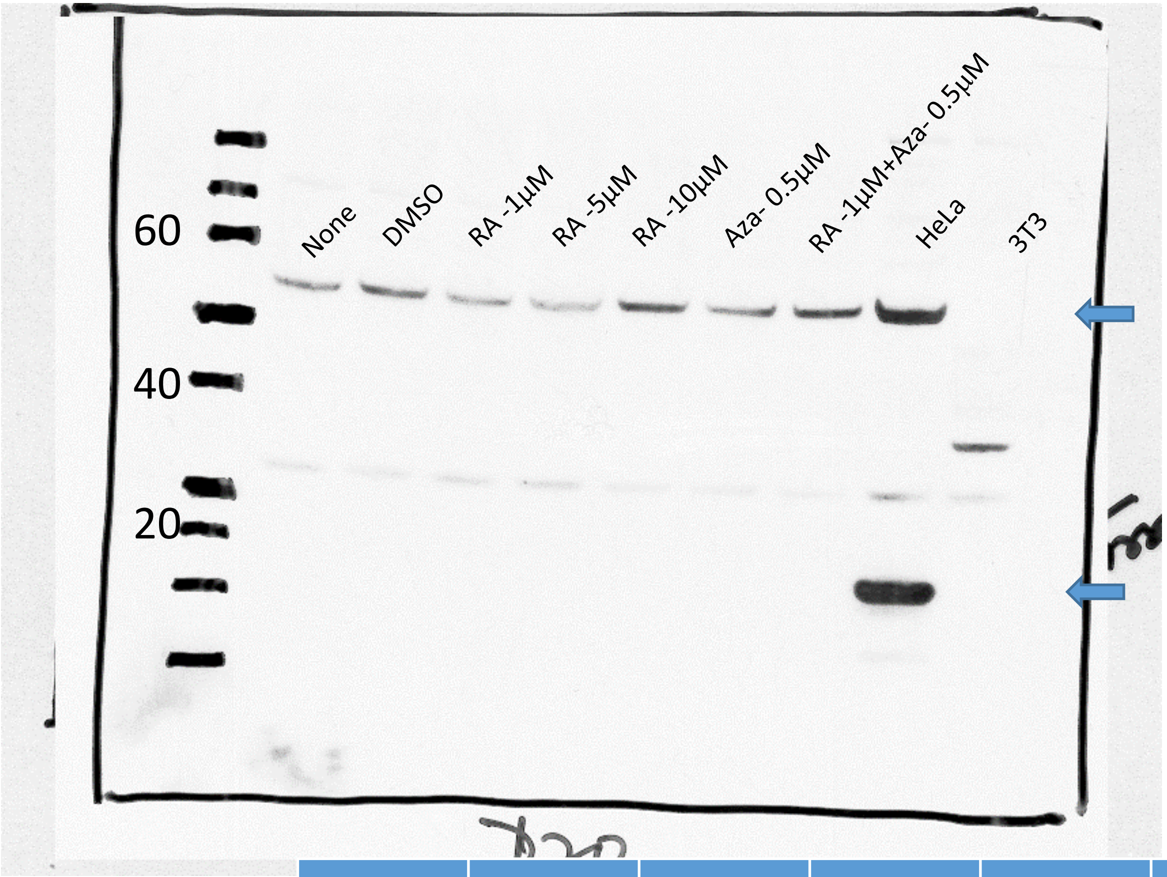


p21

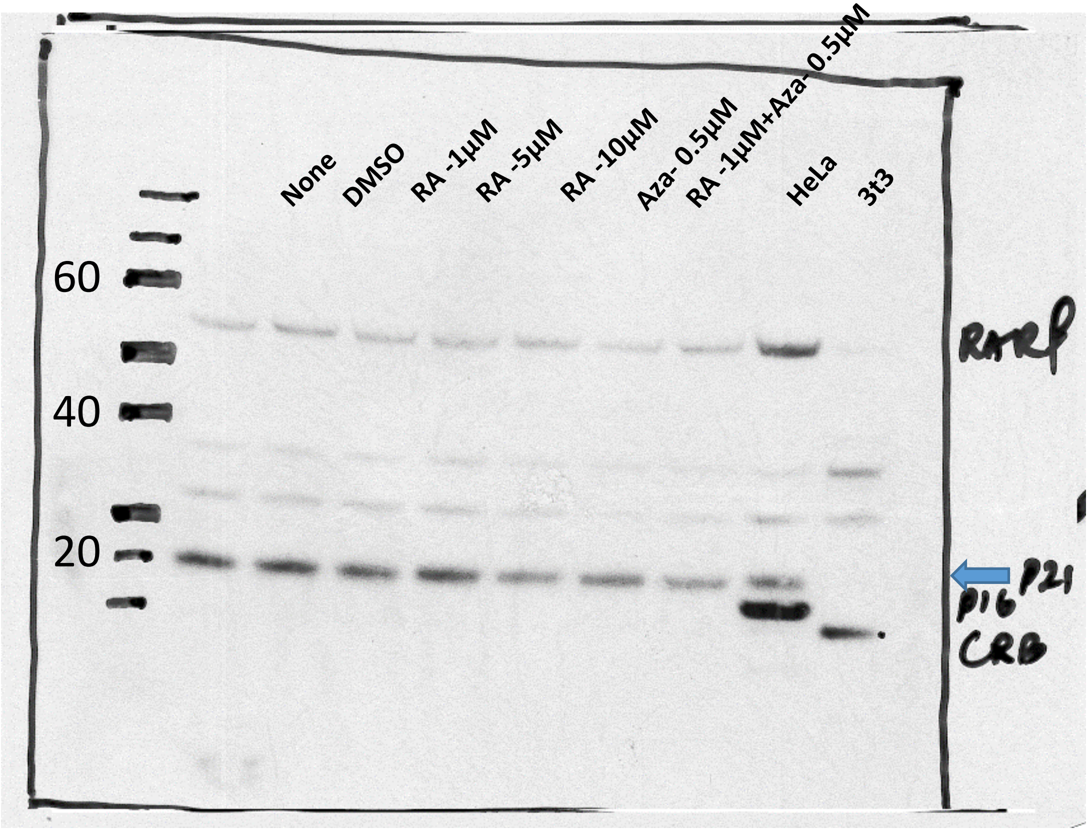


| | DMSO | No Rx | RA 1uM | RA 5uM | RA10uM | Aza 0.5uM | RA+Aza | HeLa | i3t3 |
|---|---|---|---|---|---|---|---|---|---|
| RARB | 0.50 | 0.64 | 0.36 | 0.36 | 1.19 | 0.93 | 1.41 | 3.83 | 0.02 |
| p21 | 1.17 | 1.02 | 0.93 | 1.21 | 0.85 | 1.24 | 0.81 | 1.23 | 0.02 |
| p16 | 0.02 | 0.01 | 0.02 | 0.01 | 0.02 | 0.02 | 0.03 | 6.65 | 0.02 |

# Original blots for figure 3B: D34

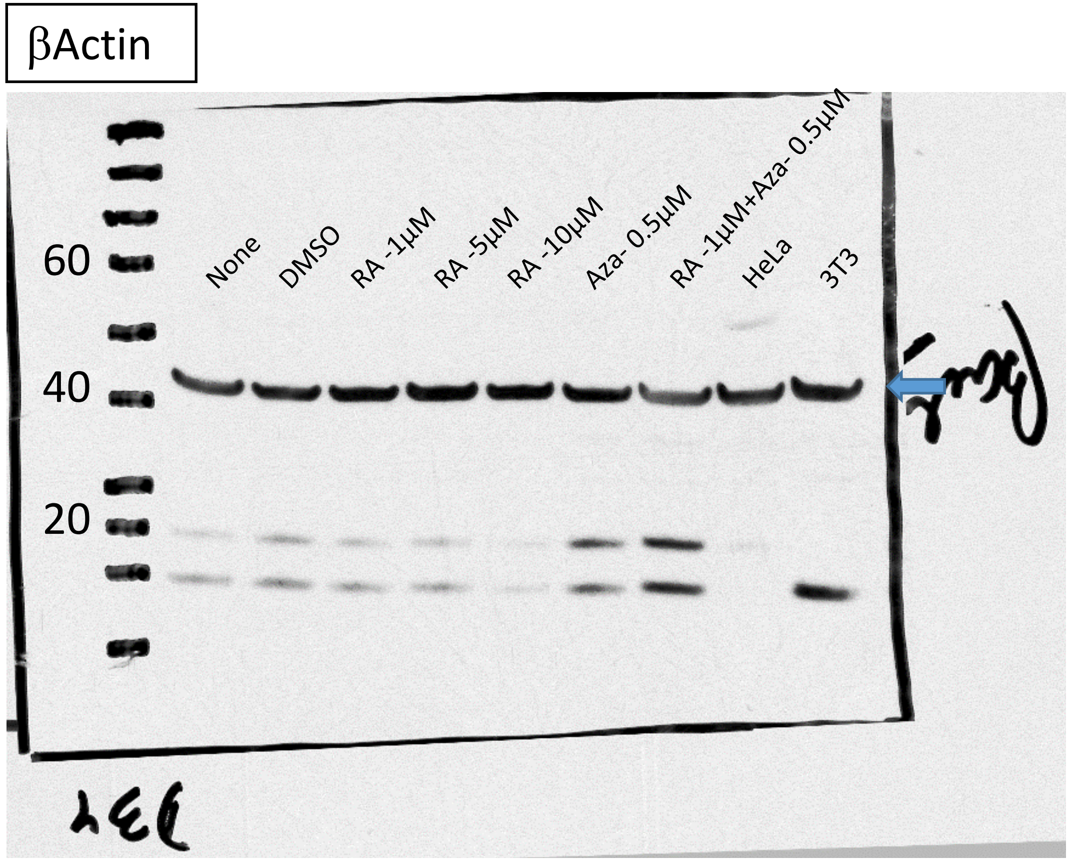

# Original blots for figure 3B: D34, with densitometry relative to B actin

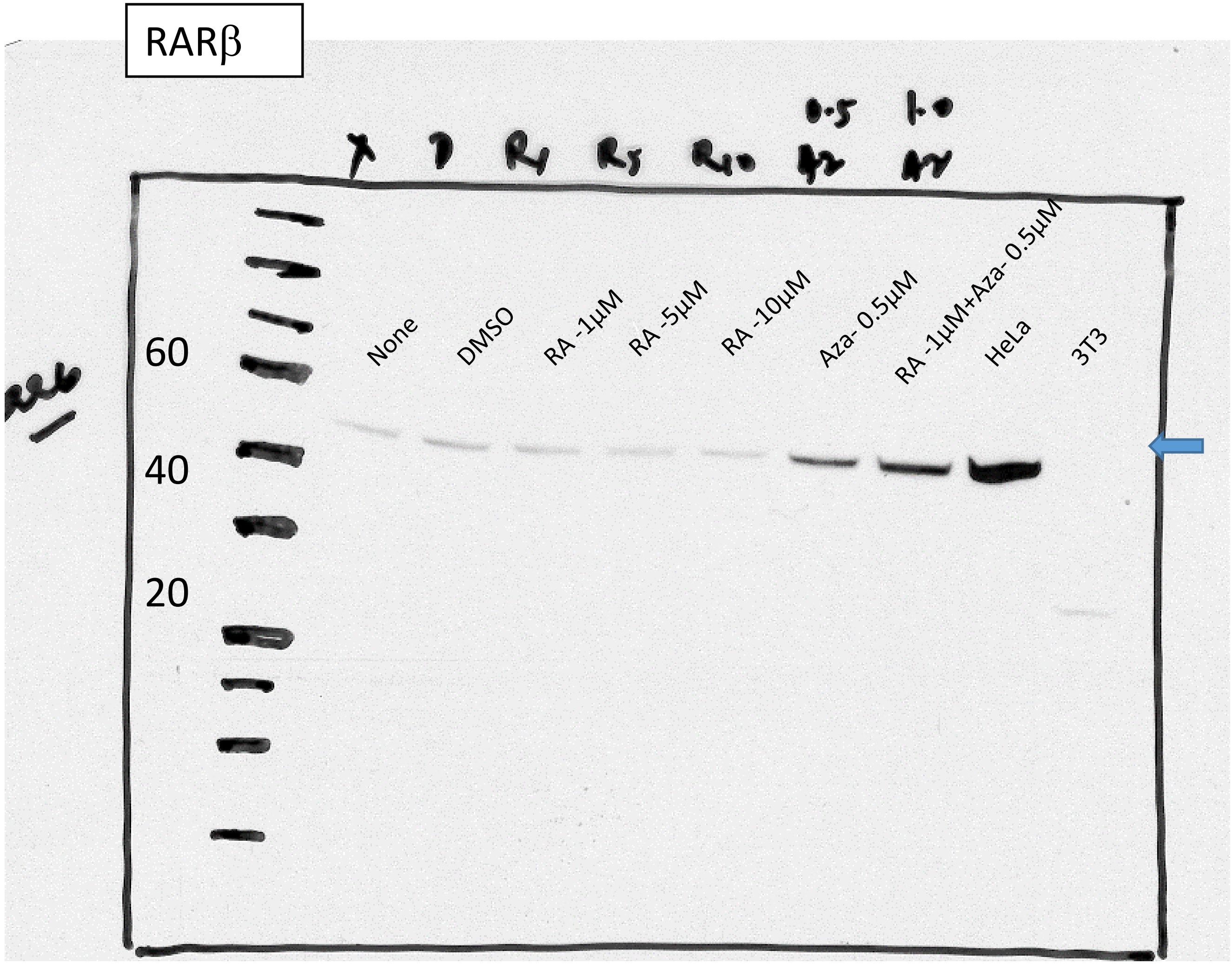


| | None | DMSO | RA 1uM | RA 5uM | RA10uM | Aza 0.5uM | RA+Aza | HeLa | i3t3 |
|---|---|---|---|---|---|---|---|---|---|
| RARB | 0.21 | 0.34 | 0.22 | 0.18 | 0.15 | 1.18 | 3.10 | 5.46 | 0.00 |

# Original blots for figure 3B: D34, with densitometry relative to B actin

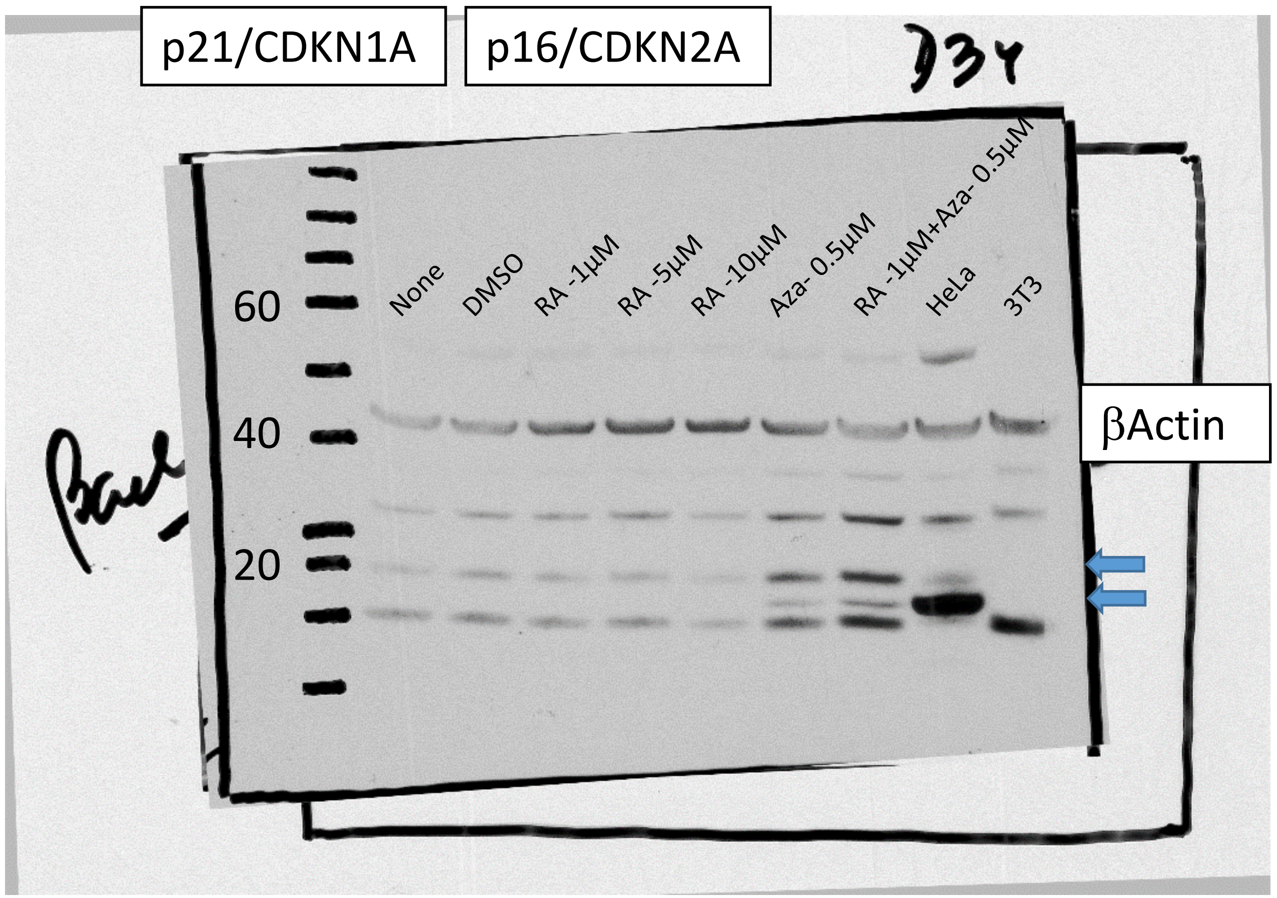


| | None | DMSO | RA 1uM | RA 5uM | RA10uM | Aza 0.5uM | RA+Aza | HeLa | i3t3 |
|---|---|---|---|---|---|---|---|---|---|
| p21 | 0.17 | 0.30 | 0.19 | 0.19 | 0.12 | 0.68 | 1.69 | 0.32 | 0.00 |
| p16 | 0.00 | 0.00 | 0.00 | 0.00 | 0.00 | 0.01 | 0.08 | 1.83 | 0.00 |

# Original blots for figure 3B: D38, with densitometry relative to B actin

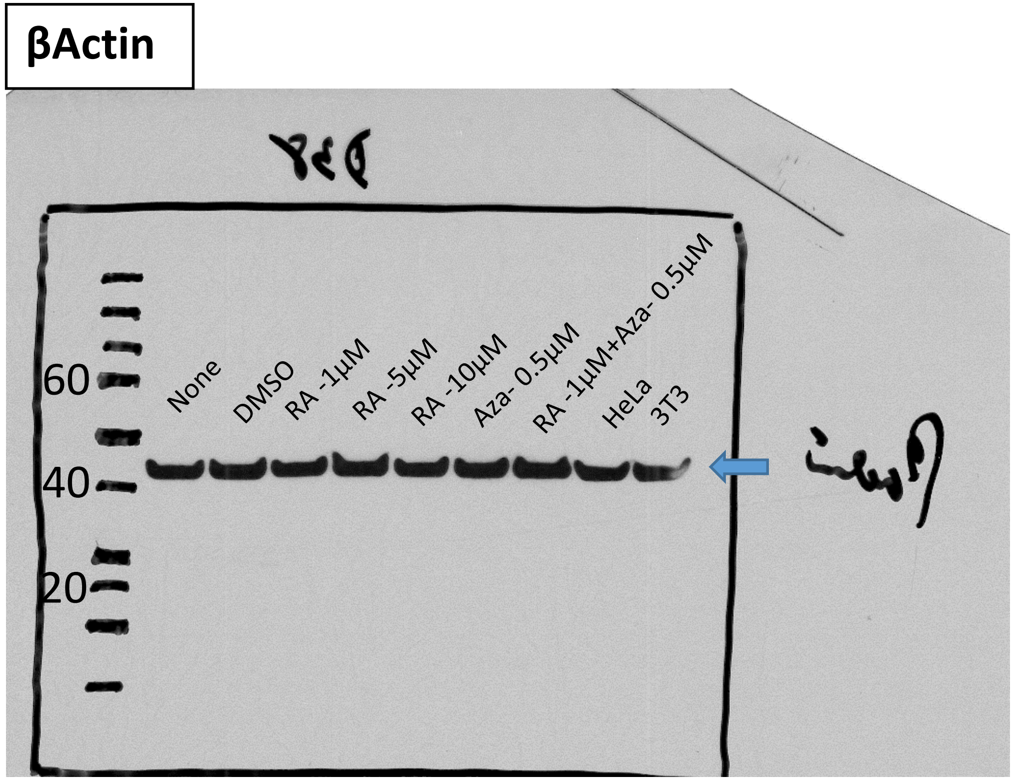


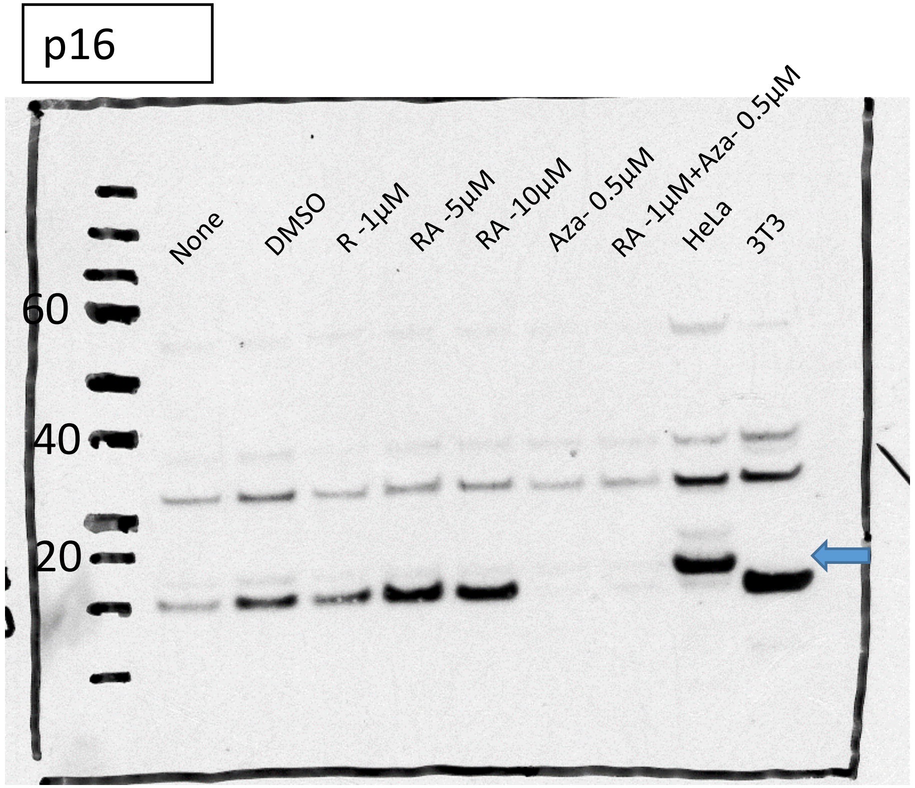


| | No Rx | DMSO | RA 1uM | RA 5uM | RA10uM | Aza 0.5uM | RA+Aza | HeLa | i3t3 |
|---|---|---|---|---|---|---|---|---|---|
| p16 | 0.02 | 0.04 | 0.00 | 0.01 | 0.00 | 0.01 | 0.01 | 0.53 | 0.00 |

# Original blots for figure 3B: D38, with densitometry relative to B actin

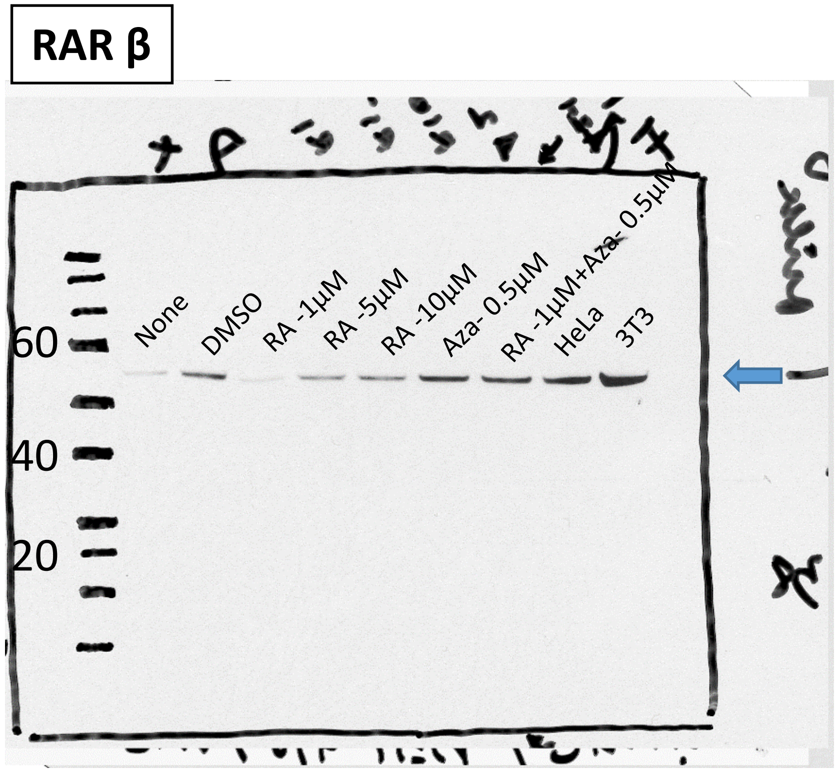


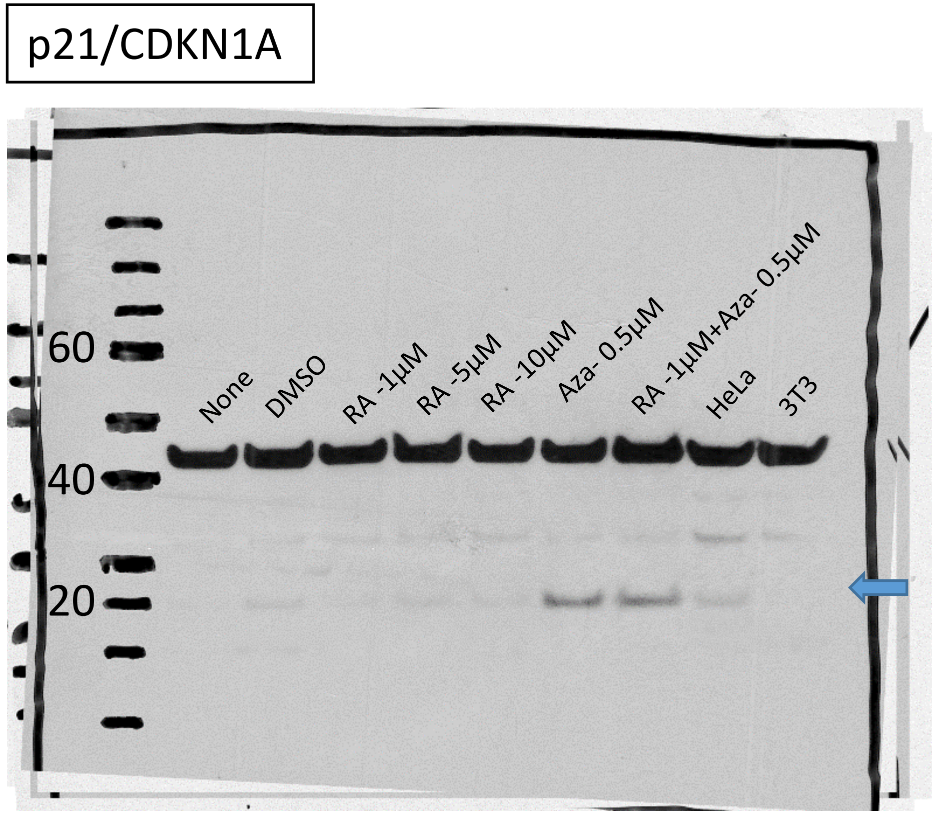


| | No Rx | DMSO | RA 1uM | RA 5uM | RA10uM | Aza 0.5uM | RA+Aza | HeLa | i3T3 |
|---|---|---|---|---|---|---|---|---|---|
| RARB | 0.15 | 0.32 | 0.16 | 0.23 | 0.30 | 0.45 | 0.38 | 0.57 | 0.84 |
| p21 | 0.03 | 0.06 | 0.04 | 0.03 | 0.02 | 0.29 | 0.26 | 0.09 | 0.02 |
